# Invasive Asian Earthworms Negatively Impact Keystone Terrestrial Salamanders

**DOI:** 10.1371/journal.pone.0151591

**Published:** 2016-05-04

**Authors:** Julie L. Ziemba, Cari-Ann M. Hickerson, Carl D. Anthony

**Affiliations:** John Carroll University, University Heights, Ohio, United States of America; Trier University, GERMANY

## Abstract

Asian pheretimoid earthworms (e.g. *Amyntha*s and *Metaphire* spp.) are invading North American forests and consuming the vital detrital layer that forest floor biota [including the keystone species *Plethodon cinereus* (Eastern Red-backed Salamander)], rely on for protection, food, and habitat. *Plethodon cinereus* population declines have been associated with leaf litter loss following the invasion of several exotic earthworm species, but there have been few studies on the specific interactions between pheretimoid earthworms and *P*. *cinereus*. Since some species of large and active pheretimoids spatially overlap with salamanders beneath natural cover objects and in detritus, they may distinctively compound the negative consequences of earthworm-mediated resource degradation by physically disturbing important salamander activities (foraging, mating, and egg brooding). We predicted that earthworms would exclude salamanders from high quality microhabitat, reduce foraging efficiency, and negatively affect salamander fitness. In laboratory trials, salamanders used lower quality microhabitat and consumed fewer flies in the presence of earthworms. In a natural field experiment, conducted on salamander populations from “non-invaded” and “pheretimoid invaded” sites in Ohio, salamanders and earthworms shared cover objects ~60% less than expected. Earthworm abundance was negatively associated with juvenile and male salamander abundance, but had no relationship with female salamander abundance. There was no effect of pheretimoid invasion on salamander body condition. Juvenile and non-resident male salamanders do not hold stable territories centered beneath cover objects such as rocks or logs, which results in reduced access to prey, greater risk of desiccation, and dispersal pressure. Habitat degradation and physical exclusion of salamanders from cover objects may hinder juvenile and male salamander performance, ultimately reducing recruitment and salamander abundance following Asian earthworm invasion.

## Introduction

The invasion of non-native species is thought to be a major driver of worldwide biodiversity loss [1]. Exotic species can negatively influence native populations through diverse direct interactions, including predation and competition [2,3]. Invasive species can also have important indirect, non-trophic effects by altering the physical environment and availability of resources as “ecosystem engineers” [4]. Although some invasive ecosystem engineers [such as *Castor canadensis* (beaver) in Cape Horn, Chile] [5], positively facilitate other biota by increasing habitat complexity [6], resource degradation is a common result of invasive ecosystem engineering [7]. Interactions between indigenous and non-indigenous species are complex, and predicting the net effects of invasion requires identifying the presence, direction, and magnitude of these direct and indirect pathways. Further, discerning the variable importance of these interactions with respect to seasonal, short-term, and long-term ecosystem processes is essential for understanding the consequences of invasion [8].

Some earthworms are cosmopolitan ecosystem engineers that have invaded and colonized all continents, except Antarctica [9]. During the Pleistocene epoch, glacial denudation of North America extirpated native earthworms from forests covered by the Laurentide ice sheet, which includes the Great Lakes region [10]. Since earthworms are limited by slow dispersal rates (0.5–1.0 km in 100 years) [11], these forests remained earthworm-free until human activities, such as European settlement in ~1700 CE, introduced exotic species [12]. Subsequently, anthropogenic activities, including composting, bait dumping, development, horticulture, and international commerce, have been the primary vectors for European, Asian, and African earthworm dispersal [13,14].

Forest floor communities lacking native earthworms are particularly vulnerable to the transformative effects of invasive earthworms [15,16]. Previously-glaciated forests evolved in the absence of ecological equivalents to earthworms. In these forests, shed organic matter gradually developed on the forest floor and formed a thick, multi-layered habitat (organic soil horizon, or O horizon) as a consequence of bacterial-dominated decomposition and nutrient cycling. This detrital duff layer traps moisture, creating a humid microhabitat that buffers inhabitants from thermal and hydric extremes, offers refugia from predation, and provides a nutrient base for the entire forest food web [17]. Rapid earthworm-driven consumption of the nutrient-dense, insulative O horizon that accumulated over thousands of years has exposed native biota to environmental conditions outside of their recent evolutionary experience.

The effects of earthworm species belonging to different ecological groups can vary based on particular trophic and microhabitat characteristics [18]. However, in general, invasive earthworms accelerate leaf litter (LL) decomposition and nutrient release, reduce the O horizon depth by consuming detritus, and alter edaphic properties via cast (excrement) production and bioturbation [19–21]. Non-indigenous earthworms reduce the diversity and abundance of native plant, microinvertebrate, amphibian, mammal, and avian species [13,18] while facilitating other exotics (i.e. invasional meltdown; exotic understory plants and deer) [22].

Asian pheretimoid earthworms (e.g. *Amynthas* and *Metaphire* spp.) are rapidly expanding their range in previously-glaciated regions of the United States [23] and causing a drastic reduction of LL layers in both disturbed and undisturbed forests following colonization [24–26]. As recent arrivals to the United States (1939) [27], the ecology and impacts of *Amynthas* and *Metaphire* spp. invasion are poorly-studied in comparison to the longer-established European genera. Some pheretimoid species, and particularly those encountered in northeastern Ohio (likely *Amynthas agrestis* and *Metaphire hilgendorfi*; hereafter referred to as “large pheretimoid earthworms” or “large pheretimoids”), live at the soil surface and construct shallow, temporary burrows in upper topsoil layers. These relatively large (65 mm average total length for *A*. *hilgendorfi*) [28] earthworms voraciously consume LL and associated microorganisms [29], and produce large quantities of casting material that changes the physical, chemical, and biotic properties of the topsoil [19,30]. The pheretimoid earthworms commonly encountered in northeastern Ohio and the focus of our study are highly active, exhibiting a unique thrashing behavior when disturbed and a serpentine style of locomotion that makes them conspicuous surface occupants. These large pheretimoid earthworms are trophically plastic [31] and some species are parthenogenetic [23], which contributes to their successful colonization and competitive domination of newly invaded environments [32]. Additionally, these large pheretimoid earthworms are negatively associated with European earthworm species in previously-glaciated forests of Ohio [25]. Finally, in contrast to most invasive European earthworm species, these large pheretimoid earthworms have an annual life cycle (Table 1). Given their aggressive colonization and combination of unique morphological, locomotory, feeding, and life history characteristics, it is likely that the effects of these large pheretimoid earthworms on the soil layers and the organisms that inhabit the forest floor community differ from those of the well-studied European species.

**Table 1:** A comparison of large pheretimoid earthworm population characteristics [33, 34] at the soil surface and *Plethodon cinereus* microhabitat occupancy [35] throughout the year in Ohio, USA.

	Fall	Winter	Spring	Summer
Pheretimoid population at the soil surface	Large adults and some juveniles	Cocoons	Hatchlings	Juveniles
Location of *P*. *cinereus* activity	Surface	Underground	Surface	Underground

There is a spatial overlap between the taxa in the spring (small earthworms are consumed by salamanders) and fall (dense populations of large pheretimoid earthworm adults may physically disturb important salamander activities).

Some invasive earthworm species co-occur with terrestrial salamanders, such as *Plethodon cinereus* (Green 1818) (Eastern Red-backed Salamander), in the LL and beneath natural cover objects (COs; rocks and logs) [36]. Studies on the behavioral interactions between woodland salamanders and invasive earthworms have largely focused on the deep-burrowing European earthworm, *Lumbricus terrestris* (Linneus 1758) (Common Nightcrawler). Red-backed salamanders will use the permanent vertical burrows of *L*. *terrestris*, which can improve salamander overwintering success and provide refugia from predators [37–40]. *Lumbricus terrestris* burrows can also provide an additional fossorial habitat for salamanders that may alleviate some of the negative impacts of LL consumption that degrades surface microhabitat [40]. The only study that has examined the effect of large pheretimoid earthworms on *P*. *cinereus* behavior found altered salamander CO use and increased salamander movement between microhabitats over time as LL was consumed by earthworms in laboratory microcosms [41]. Since large pheretimoid earthworms remove the detrital habitat of salamanders, but do not construct permanent soil burrows that serve as an alternate spatial resource, impacts of their invasion on *P*. *cinereus* populations likely differ from European species. For instance, pheretimoid-mediated consumption of the vital LL buffer may negatively impact salamanders by exposing them to harsh surface conditions and increased predation pressure. Additionally, the presence of the large, active pheretimoid individuals in scant suitable surface microhabitat may physically disrupt normal salamander activities.

Despite their small body size [*P*. *cinereus* average ~40 mm in snout-vent length (SVL) and weigh ~1 g in Ohio] [35], red-backed salamanders contribute an impressive amount of biomass to northeastern temperate forest communities (densities > 2.8 individuals per m^2^) [42]. As predators of microinvertebrates and prey for larger vertebrates, red-backed salamanders are integral trophic links in forest ecosystems and indirectly influence forest floor carbon dynamics [43,44]. *Plethodon cinereus* are lungless, and require moist substrate to respire passively through the skin [45]. Between periods of rainfall, when the dry forest floor becomes inhospitable for the desiccation-prone salamanders, natural COs are crucial refugia. Salamanders aggressively defend these valuable territories from intra- [46,47] and interspecific competitors [48–50]. Natural COs are also critical oviposition sites for these direct-developing terrestrial salamanders [35].

In the northeastern United States, populations of *P*. *cinereus* spend the majority of winter and summer underground in natural soil crevices, avoiding suboptimal surface conditions, except for mild winter days and when rainy, cool summer nights allow for sporadic nightly foraging. Since climatic factors constrain a salamander’s use of its microhabitats (underground retreats vs. LL and the substrate beneath natural COs) at different times of the year, it is possible that the habitat modification and physical effects of earthworms may shift seasonally (Table 1). Pheretimoid earthworm ontogeny may also be an important factor to consider, as smaller juvenile earthworms could provide important food sources for gravid females in the spring [51,52], but would be full-grown, and therefore too large for consumption by gravid females in the fall. Most salamander surface activity occurs in the spring and fall, during an extended breeding season [35]. Large pheretimoid earthworms may have the greatest potential to physically interfere with important salamander activities that occur both in the LL and under COs in the fall. Dense populations of large adult earthworms could disturb salamander foraging behavior, territorial defense, mating, and egg brooding (in Ohio, 6–9 weeks following oviposition in late-July) [35].

In this study, we used manipulated laboratory trials and field surveys to investigate the potential interactions between invasive pheretimoid earthworms and ecologically important terrestrial salamanders. Laboratory experiments focused on isolating the direct effects of the physical interaction between pheretimoid earthworms and *P*. *cinereus* on salamander microhabitat use and foraging behavior. In cohabitation trials, we predicted earthworms would exclude salamanders from high quality microhabitat (beneath artificial COs), resulting in salamanders using high quality microhabitat less frequently when paired with an earthworm than when alone. For the foraging trials, we posited that the presence of an earthworm would lengthen a salamander’s latency to the first attack and reduce the number of flies that a salamander is able to consume.

The focus of the field study was to evaluate the differences between populations of *P*. *cinereus* in pheretimoid earthworm-invaded and non-invaded forests of northeastern Ohio. We predicted that salamander populations in invaded forests would exhibit declines in body condition and egg production compared to those in non-invaded forests. Specifically, we hypothesized that salamander abundance, body condition, and eggs per female would decrease with pheretimoid abundance due to earthworm-mediated LL degradation and physical disturbance. Since salamanders rely on LL for camouflage from visual predators, we also expected that rates of tail breakage (a proxy for predation pressure) [53] would increase with earthworm abundance.

## Materials and Methods

### Ethics statement

All specimen collection for this study was conducted under an Ohio Department of Natural Resources collection permit (16–06) and Geauga Park District special use permit. Laboratory trials were conducted with prior approval of the Institutional Animal Care and Use Committee at John Carroll University (IACUC protocol number JCU1302).

### Laboratory experiments

Adult (snout-vent length > 35 mm; N = 120) red-backed salamanders (*Plethodon cinereus*) were collected from the Manatoc Boy Scout Camp property (MBSC; 41°13'37.2"N, 81°31'17.2" W) in Summit County, OH on 17 April 2014. European and Asian earthworms were observed throughout the field site. We recorded wet mass (g) and SVL (mm) in the laboratory for all salamanders using a digital scale and digital calipers. *Plethodon cinereus* is polymorphic, and the “striped” and “unstriped” color morphs were collected for the laboratory trials. However, we only used “striped” individuals for the microhabitat trials, as there is evidence for slightly different thermal and moisture preferences between color morphs [54,55]. Before the beginning of the experiment, salamanders were maintained individually in Pyrex dishes (11 cm diameter; 470 ml volume) with LL substrate (collected from MBSC), on a natural light: dark cycle at an average temperature of 18.5 ± 1°C. Salamanders were fed wingless *Drosophila melanogaster* (fruit flies) *ad libitum*.

Pheretimoid earthworm specimens were purchased online (The Worm Dude, California, USA). Only intact, clitellate individuals with total lengths > 65 mm were used for trials. Earthworms were housed in plastic containers (5 earthworms per container; 20 cm long x 12 cm wide x 6 cm deep) with a layer of topsoil covered by moist detritus (collected from MBSC) until the experiment began.

#### Cohabitation trials

For the cohabitation trials, a salamander and either 0 or 1 earthworm(s) were placed in a test chamber with zones of high and low microhabitat quality for 12 hours. We predicted that salamanders would use the high quality zone beneath the artificial CO more frequently when alone in the chamber than when paired with an earthworm. Each salamander (N = 36) was tested twice, once alone and once paired with an earthworm for a total of 72 trials. The order of the trials for each salamander was assigned randomly. An equal number of male and female salamanders were tested. Each earthworm was used for only one trial.

Test chambers were Thermo Scientific Nunc Square BioAssay dishes with lids (24.5 cm x 24.5 cm x 2.5 cm) [54]. Each chamber included an artificial CO (small ceramic tile; 7.5 cm x 7.5 cm x 0.9 cm) in a random corner of the dish, propped up by a 1 cm piece of plastic surgical tube (0.6 cm diameter), on top of a piece of moistened filter paper (7.5 cm diameter). To control for potential differences in humidity, 1 ml of commercially available spring water was applied to the center of the dry filter paper immediately before an animal was added to a chamber. Zone quality was defined by the level of moisture (protection from desiccation) provided. The high quality zone was the space beneath the artificial CO and on top of moist filter paper, and the low quality zone was the dry plastic bottom of the chamber. Pilot data indicated that earthworms occupied the high quality zone both when alone in the chamber and when paired with a salamander.

At the beginning of each trial salamanders were placed in a random corner of the chamber that did not contain the CO (in paired trials, earthworms and salamanders were placed in opposite corners) and the movements of the animals were video-recorded for 12 hours. Each salamander’s set of trials (alone and paired) occurred on consecutive days. We sampled each 30 minute interval (N = 24) of the salamander’s 12-hour videos to establish how frequently the individual used each zone when alone and when paired with an earthworm. The video was paused and the salamander’s location was determined based on which zone contained > 50% of the salamander’s trunk or 50% of the salamander’s trunk and its head. Three digital video cameras (Sony HDR-CX240 Full HD Handycam camcorders) recorded 12 trials per sampling date. Each video camera filmed a group of chambers, with two replicates of each treatment (salamander only and paired) arranged in a randomized block. Trials were conducted on 9–15 August 2014 from 2100–0600 hours, under dim red illumination (Safelight B, 15·W bulb; Kodak, Rochester, NY, USA) [56] and from 0600–0900 hours under ambient illumination to approximate a natural night: day lighting regimen.

#### Foraging efficiency trials

The foraging efficiency trials evaluated the effect of pheretimoid earthworms on salamander foraging behavior. Salamanders were tested in the same chambers used in the cohabitation experiment with either 0 or 1 earthworm(s). The chamber substrate was a saturated paper towel covered by a single layer of moistened leaves (dried in an oven for 48 hrs to remove invertebrates). Each animal was only used for one trial. Salamanders were ranked by mass and distributed evenly among treatments. Salamander morphs (striped and unstriped) and genders were also distributed evenly among treatments. Since light intensity has been shown to alter foraging efficacy in nocturnal amphibians [57], all trials were under dim red light (Safelight B, 15·W bulb; Kodak, Rochester, NY, USA) [56]. Before the beginning of a trial, food was withheld from the salamander for 7 days to standardize the hunger level among individuals. Trials were conducted on 16–23 June 2014 during the day.

At the beginning of each trial, a salamander and either 0 or 1 earthworm(s) were placed in opposite corners of the chamber and allowed to acclimate for five minutes. During the acclimation period, the salamander was covered by an opaque petri dish (5 cm diameter). Following acclimation, 15 wingless *D*. *melanogaster* were randomly added to the chamber and the acclimation cover was removed. Salamander foraging behavior was observed and timed until the first fly was captured (latency to first attack). The trials lasted for 20 minutes and the number of flies consumed by the conclusion of the trial was recorded. All earthworms were killed immediately after the experiments. Salamanders were either euthanized via immersion in MS222 or they were used in additional experiments. No salamanders were released, due to the risk of disease transmission to natural populations. All salamanders were deposited into the Cleveland Museum of Natural History.

### Field experiment

To evaluate the effect of pheretimoid earthworm invasion on the fitness of *P*. *cinereus*, measures of salamander abundance, body morphometrics, predation pressure, and reproductive effort were compared between populations from “pheretimoid earthworm-invaded” and “non-invaded” forests. We predicted that salamander populations at pheretimoid earthworm-invaded sites would have lower abundances, reduced body condition, greater rates of tail breakage, and fewer eggs per gravid female than those at non-invaded sites. We also hypothesized that pheretimoids would exclude salamanders from COs, resulting in less co-occurrence of earthworms and *P*. *cinereus* beneath the same CO than expected by chance.

#### Site classification

All field sites were within previously-glaciated, beech-maple hardwood forests in northeastern Ohio (S1 Table, S1 Fig). Vegetation, detrital fauna, and land use history were similar for all field sites. These sites were classified as pheretimoid earthworm-invaded or non-invaded based on the presence of earthworm individuals (or earthworm casting material) and LL quality (modified from a European earthworm invasion rapid assessment method) [58]. Pheretimoid earthworms were identified based on the presence of an annular clitellum, “jumping” behavior, and caudal autotomy [59]. Current taxonomic flux regarding the classification of pheretimoid earthworm species [60] did not allow for the resolution of our earthworms to species. However, individuals belonging to *Amynthas agrestis* and *Metaphire hilgendorfi* have previously been identified at these field sites [25].

The leaf litter quality (LL score) of each site was evaluated by sampling four random 1 m^2^ quadrats. Individual quadrats were ranked on a scale of 1–3 and then added together to obtain the LL score for each site. A rank of 1 indicated a low quality forest floor, with a thin organic horizon composed of leaf-fall from only the previous year. A rank of 3 indicated a high quality forest floor, with a thick, multi-layered organic horizon with 3+ years of leaf-fall. A rank of 2 indicated intermediate forest floor quality. The maximum LL score that a site could receive was a 12 (a rank of 3 at all four sampled quadrats; an intact, healthy forest floor), while the minimum LL score a site could receive was a 4 (a rank of 1 at all four sampled quadrates; a highly disturbed, scant forest floor). Sites were classified as “non-invaded” if there were intact, healthy LL layers (LL score > 9) and low European earthworm burden (< 5 individuals found from raking through LL during forest floor quality assessment and < 50 individuals found at the site in total).

#### Animal surveys

Salamander and earthworm data were collected during the day (0800–1700 hrs) from 18 September–25 October 2014, during the fall breeding season for *P*. *cinereus*. We visited the localities in semi-random order, alternating between pheretimoid earthworm-invaded and non-invaded forests to control for the effect of differences in precipitation on salamander detection (during and immediately following rainfall, salamanders will leave COs to forage in the LL) [61]. Forests that contained both pheretimoid earthworm-invaded and non-invaded areas were surveyed on consecutive days (The West Woods and Holden Arboretum; S1 Table).

To obtain measures of earthworm (pheretimoid and surface-dwelling European spp.) and salamander abundances, we flipped the first 100 natural COs encountered at the site (within an area up to 0.5 hectares) and recorded how many individuals were found. Only rocks or logs that were 25–50 cm^2^ (containing no space for refuge from interaction) were flipped to ensure that we were measuring actual co-occurrence of pheretimoid earthworms and *P*. *cinereus*. We also recorded if salamanders were alone or sharing the CO with a pheretimoid earthworm. Salamander SVL, wet mass, eggs (counted through the ventral body wall), and age class [juvenile (sexually immature): SVL < 35 mm, adult: SVL > 35 mm] were recorded. Tail breakage (or regrowth) was recorded as a proxy for predation pressure. After the initial 100 COs were flipped to determine a standardized salamander abundance per site, we continued to flip any CO encountered until body morphometrics were collected for at least 50 total salamanders per site.

### Statistical analysis

Before all analyses, the Shapiro-Wilk and Levene’s tests were performed to determine if the data met the test assumptions of normality and homoscedasticity. When necessary, appropriate remedial transformations and weighting (by the inverse variance) [62] were applied to non-normal and heteroscedastic data. For the microhabitat trials, two-tailed, paired t-tests were used to evaluate the effect of pheretimoid earthworms on salamander microhabitat use. For the foraging trials, the effect of earthworm presence on a salamander’s latency to attack and the number of flies consumed by the end of the trial were analyzed using a randomized complete block ANOVA. The main effects were earthworm presence (absent or present), gender (male or female), and morph (striped or unstriped). There were no significant two-way interactions, so the models were decomposed to include only the main effects.

Univariate linear regression was used to compare the relationship between pheretimoid earthworm abundance and variables of interest (LL score, European earthworm abundance, salamander abundance, number of eggs/female salamander, and tail breakage). For pheretimoid earthworm-invaded sites, the probability of *P*. *cinereus* and pheretimoid earthworm co-occurrence beneath a CO was evaluated using a chi-square test with four categories (salamander present, earthworm present, earthworm and salamander present, neither present). Chi-square analyses were not completed for individual pheretimoid earthworm-invaded sites because the data did not meet the test assumptions. Body condition was calculated for juvenile, male, and female salamanders using the scaled mass index [63]. Nested ANOVA analyses were used to compare salamander body condition between populations at invaded and non-invaded sites. For all analyses, α = 0.05. Analyses were completed using SPSS v. 21.

## Results

### Laboratory experiments

**Fig 1:**
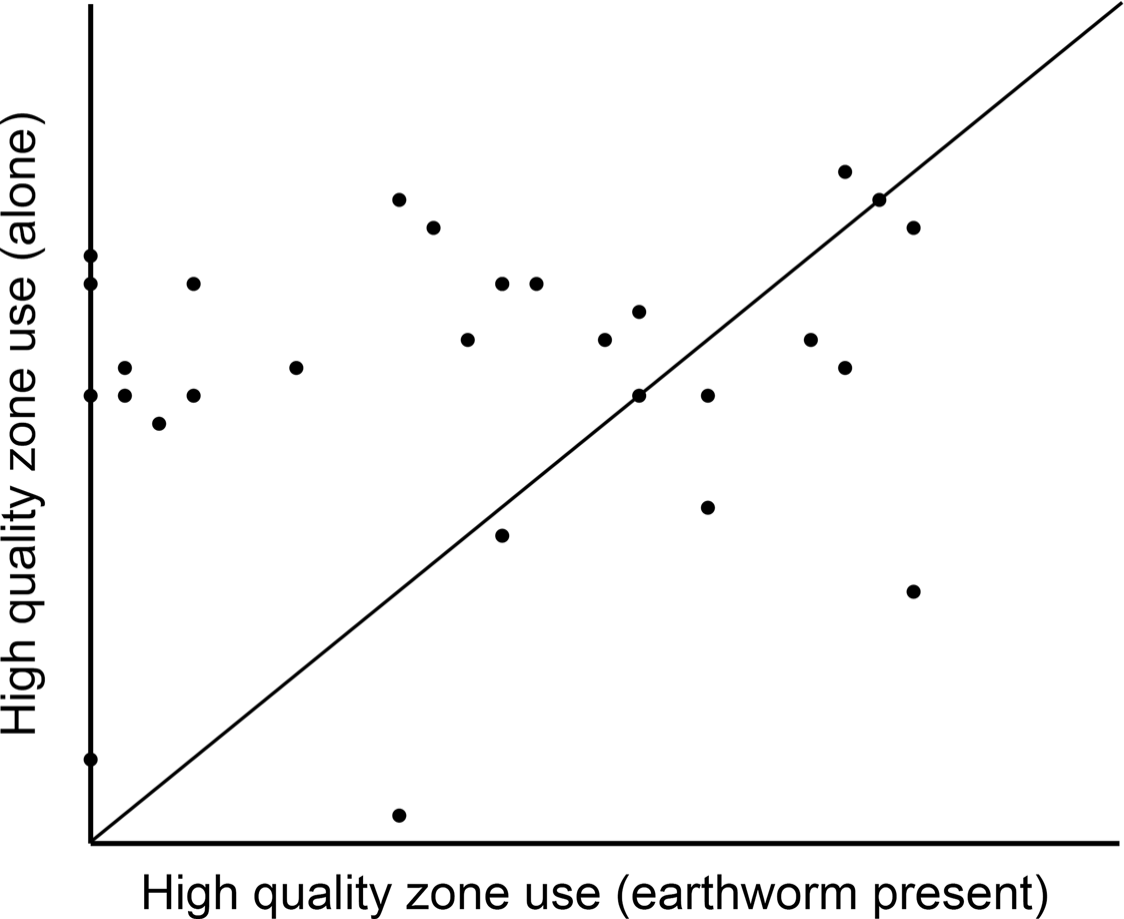
Equal probability plot for high quality (HQ) microhabitat use when salamanders (*Plethodon cinereus*) were alone or paired with pheretimoid earthworms. The diagonal line indicates no difference in a salamander’s HQ zone use when they were alone vs. paired with an earthworm. Points above the line represent salamanders that used the HQ zone more often when alone, while points below the line represent salamanders that used the HQ zone more often when paired with an earthworm.

Seven salamanders never used the CO during the 24-hour microhabitat trials when alone in the chamber, so they were excluded from the analyses. We found a significant effect of earthworm presence on the quality of microhabitat used by salamanders (*t*_28_ = -3.4, P = 0.002; Fig 1). Salamanders used low quality microhabitat ~34% more when paired with an earthworm than when alone.

**Fig 2:**
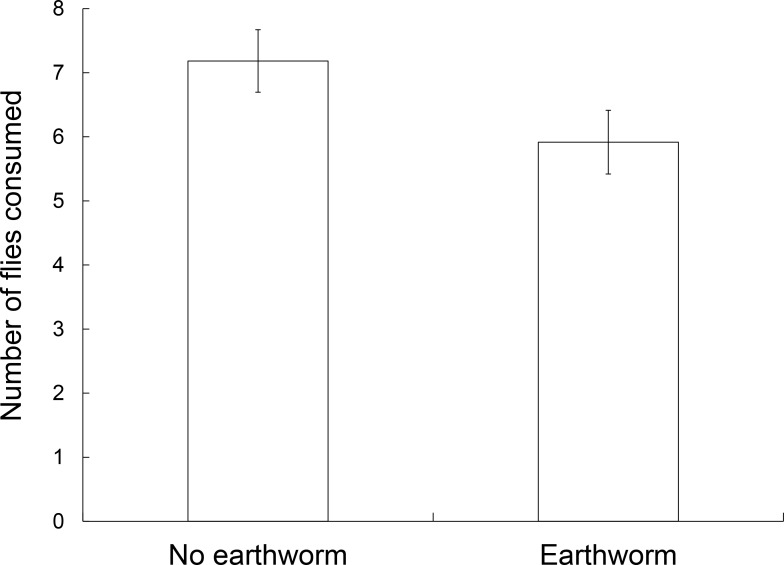
The effect of pheretimoid earthworm presence on the number of flies consumed in salamander (*Plethodon cinereus*) foraging efficiency trials. Salamanders consumed fewer flies in the presence of earthworms than when alone (*F*_1,109_ = 4.187, P = 0.043). The bars represent standard error.

Eleven salamanders did not attack a fly during the 20 minute foraging trials (N = 120) and eight of those salamanders were in trials with earthworms. There were significant effects of earthworm presence (*F*_1,109_ = 4.187, P = 0.043; Fig 2) and salamander gender (*F*_1,109_ = 11.656, P = 0.001) on the number of flies consumed. On average, salamanders in trials with earthworms (mean ± SE, 5.92±0.5) ate ~19% fewer flies than those not paired with an earthworm (7.18±0.49), and males (7.54±0.47) ate ~31% more flies than females (5.53±0.5), regardless of treatment. There was no significant effect of earthworm presence (*F*_1,109_ = 1.830, P = 0.179) on a salamander’s latency to first attack. Yet, several salamanders were observed following the earthworm around the chamber, displaying aggressive postures (all trunk raised [64]), and even biting at the earthworms throughout the trials (S1 Video).

### Field experiment

No pheretimoid earthworms were found at sites classified as “non-invaded” (N = 7), while a total of 133 individuals were found at “pheretimoid earthworm-invaded” sites (N = 6). Pheretimoid abundance at invaded sites ranged from 3–50 individuals (S1 Table). Although only surface-dwelling European earthworms would have been detected with this sampling design, very few of these individuals were found at non-invaded (36) and pheretimoid earthworm-invaded sites (3). There was a significant negative linear relationship between pheretimoid earthworm abundance and LL score (R^2^ = 0.886, *F*_1,11_ = 85.634, P < 0.0001). Thinner, lower quality LL layers were associated with higher abundances of pheretimoid earthworms. At sites where earthworms were detected, there was also a significant negative association between abundances of pheretimoid and surface-dwelling European earthworms (R^2^ = 0.544, *F*_1,8_ = 9.562, P = 0.015).

**Fig 3:**
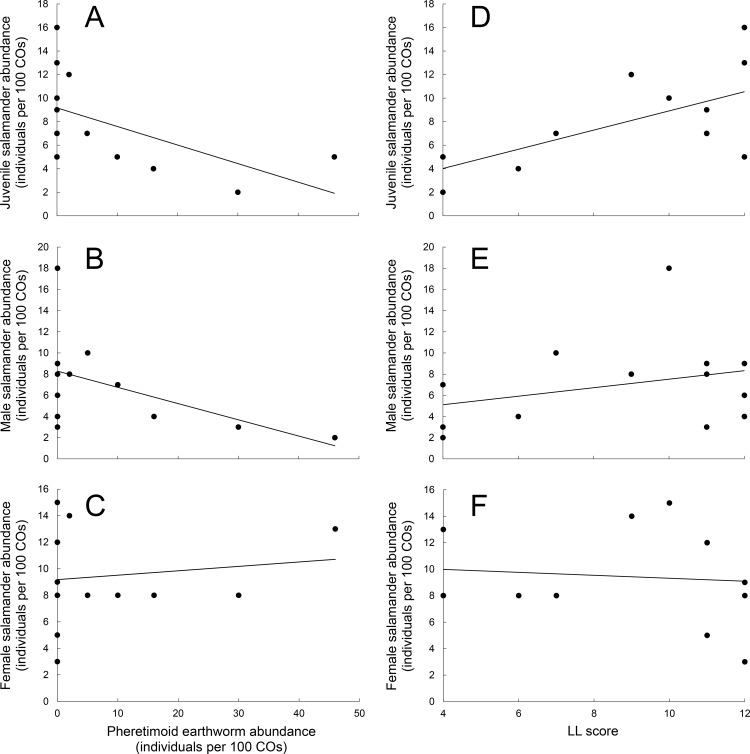
The relationship between pheretimoid earthworm and *P*. *cinereus* as measured by earthworm abundance and LL score. Juveniles were significantly associated with earthworm abundance (A) and LL score (D). There was a negative relationship between earthworm and male salamander abundance (B) but no relationship between male salamanders and LL score (E). Female salamanders were neither associated with worm abundance (C) or LL score (F). Asterisks indicate statistically significant results.

Red-backed salamanders and pheretimoid earthworms co-occurred beneath COs ~60% less often than expected (*χ*^2^ = 25.86, df = 1, P < 0.0001) and salamanders and earthworms were found alone more often than expected (25% and 21% more often, respectively). Juvenile (R^2^ = 0.501, *F*_1,11_ = 11.064, P = 0.007) and male (R^2^ = 0.301, *F*_1,11_ = 4.738, P = 0.052) salamander abundances were negatively correlated with pheretimoid earthworm abundance (Fig 3). However, there was no association between pheretimoid earthworm abundance and overall (R^2^ = 0.179, *F*_1,11_ = 2.405, P = 0.149) or female (R^2^ = 0.018, *F*_1,11_ = 0.206, P = 0.658) salamander abundance (Fig 3). It is possible that negative relationships between salamander and earthworm abundance might emerge from seasonal shifts in surface activity of salamanders and mortality of earthworms. LL score is the result of earthworm activity over multiple years and should be independent of temporal factors that might be at play within the short time scale of our field study. We examined relationships between LL score and salamander abundance and found similar patterns, except that LL score was not significantly related to abundance of male salamanders (juvenile salamanders, *F*_1,11_ = 10.469, P = 0.008; male salamanders *F*_1,11_ = 1.866, P = 0.199; all salamanders, *F*_1,11_ = 1.925, P = 0.193; female salamanders *F*_1,11_ = 0.187, P = 0.678). Pheretimoid earthworm invasion had no effect on juvenile (*F*_1,6.097_ = 1.719, P = 0.262), adult male (*F*_1,6.010_ = 1.495, P = 0.319), or gravid female (*F*_1,6.008_ = 0.0935, P = 0.531) salamander body condition. There was no effect of pheretimoid earthworm abundance on the number of eggs/female salamander (R^2^ = 0.064, *F*_1,11_ = 0.748, P = 0.41) or salamander tail breakage (R^2^ = 0.028, *F*_1,11_ = 0.32, P = 0.58).

## Discussion

Invasive Asian pheretimoid earthworms are drastically altering the resource quality and indigenous biodiversity of detrital communities in the United States [25,32]. Previously-glaciated forests that lack native earthworms are particularly vulnerable to habitat degradation following exotic earthworm invasion [15]. Some pheretimoid earthworms live at the soil surface and spatially overlap with *Plethodon cinereus*, an ecologically important forest floor amphibian [23]. The purpose of this study was to investigate the interactions between aggressive, understudied invasive earthworms and a native keystone salamander. We hypothesized that the physical presence and ecosystem engineering of pheretimoid earthworms would disturb normal salamander activities and reduce salamander fitness. Consistent with our predictions, pheretimoid earthworms excluded salamanders from beneath COs and reduced prey capture in laboratory trials. A comparison of *P*. *cinereus* populations from pheretimoid earthworm-invaded and non-invaded forests of northeastern Ohio also supported our hypothesis because there was reduced juvenile and male salamander abundance at invaded sites, as well as fewer instances of pheretimoid earthworm-salamander co-occurrence beneath COs than expected. In contrast to our predictions, there was no evidence to support an effect of pheretimoid earthworm invasion on salamander body condition, egg production, or tail breakage.

Natural COs are vital resources for terrestrial salamander success because they provide protection from desiccation when the forest floor is dry, as well as dependable access to prey and mates [65]. Red-backed salamanders are territorial and aggressively defend the space beneath rocks and logs from con- and heterospecifics [47,49,50,66]. Data collected from laboratory trials and field observations indicate that pheretimoid earthworms exclude *P*. *cinereus* from valuable CO microhabitat. These findings are consistent with a previous laboratory microcosm experiment that found altered *P*. *cinereus* microhabitat use in the presence of pheretimoid earthworms and increased importance of CO microhabitat following earthworm-mediated LL consumption [41]. In the present study, salamanders used low quality microhabitat (dry areas outside of the CO) significantly more often when paired with earthworms than when alone in laboratory trials. Correspondingly, in the field, *P*. *cinereus* and pheretimoid earthworms shared COs significantly less than expected by chance. Although pheretimoids may physically displace salamanders, there is evidence that *Amynthas agrestis* produces distasteful skin secretions when perturbed that may also deter salamanders [67]. Yellow, mucous-like earthworm secretions were found in several chambers following microhabitat trials. Since red-backed salamanders have well-developed chemoreception via nasolabial grooves [68], potential chemical-based avoidance of pheretimoid earthworm-occupied microhabitat should be investigated. As surface-dwelling animals that require moist substrate to respire through their skin, earthworms and *P*. *cinereus* are limited by natural CO availability. Domination of surface spatial resources by pheretimoid earthworms at our field sites in NE Ohio may result in negative fitness consequences for salamanders, since they could be more prone to desiccation, have reduced access to microinvertebrate prey, and fewer mating opportunities without the maintenance of stable territories.

Red-backed salamanders use both ambush (vision-based, under bright or dim lighting) and active (chemosensory-based, in the dark) predation methods to opportunistically consume LL microinvertebrates [69]. In laboratory foraging trials, the presence of large pheretimoid earthworms significantly reduced the amount of prey captured by salamanders, but did not affect the latency to a salamander’s first attack. A salamander’s latency to first attack seemed to be heavily influenced by the random initial proximity of a prey item to the salamander, and therefore may not be the best measure of predation efficiency. The presence of an earthworm reduced salamander prey capture by 19% which could result in a substantial energy loss for salamanders under natural conditions. The reduced prey capture in trials with pheretimoid earthworms may be due to visual impediment (large earthworms block salamanders from locating or attacking prey).

We observed that some salamanders followed the earthworm around the chamber and displayed aggressive behaviors (look toward, all trunk raised, and biting) [64] instead of foraging. Aggression toward earthworms could accrue an energy cost for salamanders (expensive aggressive activity combined with lost foraging time) [64]. Since there is evidence that male red-backed salamanders are more territorial than females [70], we expected males to exhibit territorial responses toward pheretimoid earthworms more often or for a longer duration than females, resulting in reduced prey capture [46]. However, we found that male salamanders consumed significantly more flies than females, which is particularly surprising, since previous studies on *P*. *cinereus* found either no difference in foraging between genders [71], or superior foraging by females (in terms of prey size and quantity) [72]. It is unclear why females consumed fewer flies than males in this study. Behavioral trials to quantify salamander aggression and escape effort in the presence of earthworm invaders may provide insight into *P*. *cinereus*-pheretimoid earthworm competition dynamics and clarify the difference in foraging responses between genders we found.

Consistent with previous studies, forests invaded by large pheretimoid earthworms had thinner, lower quality LL layers and an abundance of casting material covering the soil surface [19,24,26]. Since red-backed salamanders rely on a thick detrital matrix to buffer extreme thermal and hydric surface conditions, provide refugia from predators, and gain access to microinvertebrate prey [71], we expected reduced salamander fitness and abundance in pheretimoid earthworm-invaded forests. Despite the significant negative effect of pheretimoid earthworms on LL quality, we found no difference in salamander body condition between populations at invaded and non-invaded sites. However, juvenile and male salamander abundances significantly declined with increasing pheretimoid earthworm abundance. These results are consistent with past studies that reported declines in red-backed salamander populations following exotic earthworm-mediated LL loss [73]. However, this study is the first to find different responses among age/sex classes of salamanders. We found that, unlike that of juveniles and males, female salamander abundance was not affected by pheretimoid earthworm invasion.

Overall, red-backed salamanders have low vagility (dispersal less than 80 m/year) [74], small home ranges (5–25 m^2^) [75] and exhibit site tenacity to CO territories from year-to-year [76]. Dispersal in *P*. *cinereus* is thought to be male-biased, occurring both during the juvenile stage (allowing smaller salamanders to avoid resource competition with larger adults) [77], and after maturation (allowing non-resident males to avoid mate competition with territorial residents) [78]. The absence of a LL buffer could be a significant dispersal barrier to desiccation-prone terrestrial salamanders. It is possible that juveniles and males in pheretimoid earthworm-invaded forests must disperse farther, through habitat lacking LL cover to successfully find territories unoccupied by conspecifics or earthworms. Thus, juvenile and male salamander success could be disproportionally reduced in pheretimoid earthworm-invaded forests because of increased risk of desiccation or predation during dispersal.

Compared to adult salamanders, juveniles have a higher surface area: volume and therefore experience greater physiological constraints on surface activity because they desiccate more easily [79]. Therefore, juvenile salamanders may not be able to reach unoccupied spatial resources, and subsequent competition with superior adults could reduce recruitment rates [80]. Since there was no difference found in the number of eggs per female salamander at pheretimoid earthworm-invaded vs. non-invaded sites, it is likely that the decline in juvenile abundance is related to the success of individuals following oviposition. There has been no research regarding the effects of exotic earthworm invasion on salamander brooding or hatchling success. Earthworm casting material that covers the soil surface has different chemical, physical, and biotic characteristics than topsoil [19,81]. The pellet-like, granular casts of large pheretimoid earthworms have higher pH, retain less moisture, and contain different microflora than soil [25,82], so it is possible that salamander eggs may develop differently in pheretimoid earthworm-invaded forests. The large pheretimoid earthworms at our field sites do not construct permanent burrows, and it is unknown if there are animal burrows or natural crevices present in earthworm casting. Since red-backed salamanders do not excavate their own burrows for oviposition, but instead rely on burrows made by other animals and natural crevices in the soil beneath natural COs [35,83], a lack of appropriate oviposition sites could reduce reproductive success. Additionally, large pheretimoid earthworms could physically disturb brooding female salamanders, compromising their efforts to protect the eggs from desiccation, predation, and lethal microbial infection [35,84]. More research concerning salamander egg development, egg brooding, and recruitment in pheretimoid earthworm-invaded forests is needed.

The differing effects of pheretimoid earthworm invasion on the abundances of adult male and female salamanders at our field sites may be due to variable surface activity between genders. Field data indicated that male salamander abundance decreased with increasing pheretimoid earthworm abundance, but there was no relationship between pheretimoid earthworm invasion and female salamander abundance. In Ohio, the greatest surface activity for red-backed salamanders occurs during the breeding season (late fall–early spring) [35], when males aggressively vie for access to mating partners at the soil surface [85]. Male *P*. *cinereus* are reproductive annually, whereas females only reproduce once every two years (due to the high cost of egg production) [86]. Since males are actively searching for mates at the soil surface every year (and particularly in the fall when large adult pheretimoid earthworm abundance is greatest), there is a greater overlap with earthworms, which could explain the unique negative consequences for males found in this study.

The lack of data concerning large pheretimoid earthworm invasion history in northeastern Ohio forests was a potential drawback of this study. Although pheretimoid earthworm abundance and LL score may be used as crude measures of how long pheretimoid populations have been established, there were no data available confirming a site’s invasion history [87]. It is possible that some signals of pheretimoid earthworm invasion on salamander fitness (body condition and predation pressure) could have been obscured by differences in the amount of time that the earthworms have been established at the invaded sites. There are conflicting findings concerning the effect of large pheretimoid earthworms on microinvertebrate diversity and abundance [18,25,73], so it is possible that short-term vs. long-term impacts of earthworm invasion may differently influence LL arthropods. For instance, initial earthworm invasion rapidly releases nutrients bound up in detrital layers, potentially bolstering microinvertebrates, but unused nutrients will be leached, leading to drastically reduced resources over time [18,25]. Eventually, when pheretimoid earthworms remove the entire organic horizon, microinvertebrate abundances will decline and salamanders may have to expend more energy to locate prey. Red-backed salamanders have low metabolic rates, are adapted for climate-based shifts in prey availability, and regularly experience periods of starvation (during egg brooding, dispersal, and inhospitable surface conditions) [88]. So, it is also possible that the effects of pheretimoid earthworm-mediated LL degradation on microinvertebrate prey populations at the sites used for this study may not yet be strong enough to significantly alter salamander body condition. Further, despite low detection of surface-dwelling European earthworms at the sites used for this study, between-site differences in the presence of other invasive species (flora and fauna) might have confounded the ability to obtain a clear measure of pheretimoid invasion on salamander populations. Surveying efforts to track large pheretimoid earthworm population establishment and subsequent ecological impacts on an accurate temporal scale are essential for the ability to quantify short term vs. long-term effects of pheretimoid invasion [18,25].

## Conclusions

This is the first study to identify disparate effects of earthworm invasion on age classes and genders of red-backed salamanders. We found that pheretimoid earthworm invasion negatively impacts red-backed salamander populations in northeastern Ohio, resulting in declines in juvenile and male salamander abundances. The negative spatial association of salamanders and large pheretimoid earthworms in the field was consistent with results from laboratory trials, in which earthworms excluded salamanders from high quality microhabitat beneath COs. Additionally, salamander prey capture was significantly reduced in the presence of large pheretimoid earthworms. The removal (LL) and domination of spatial resources (natural COs) in forest floor communities by large pheretimoid earthworms may pose a significant threat to the social, trophic, and physiological success of red-backed salamanders. Although *P*. *cinereus* populations are quite robust throughout the species’ range, declines in red-backed salamanders could have important trophic ramifications for detrital communities. Removing keystone salamander predators of LL microinvertebrates could have ecosystem-level effects on nutrient cycling and carbon retention [44].

Since large pheretimoid earthworms have also invaded and altered communities of unglaciated forests in the United States [32,33], there is also cause for concern for populations of less-abundant, ecologically similar forest floor plethodontids that may be likewise negatively affected by pheretimoid earthworm invasion. Populations of rare, endangered, or endemic plethodontid species like *P*. *hubrichti* (Peaks of Otter Salamander), *P*. *nettingi* (Cheat Mountain Salamander), and *P*. *shenandoah* (Shenandoah Salamander) may not be as resilient to effects of pheretimoid earthworms and more prone to extirpation following invasion. The potential for interactive effects of pheretimoid earthworm invasion, anthropogenic stressors, and climate change may further threaten these sensitive forest floor amphibians. As large pheretimoid earthworms rapidly expand their ranges throughout the United States [34], understanding how native forest floor biota are impacted will be vital for monitoring detrital community stability and informing conservation priorities.

## Supporting Information

S1 Fig Map of field sites in northeastern Ohio. Study site localities in northeastern Ohio. Areas of Ohio denuded by the Laurentide ice sheet during the Wisconsinan glaciation (containing very few North American earthworms) are shaded gray. Non-invaded sites were: Rocky River (RR), Mill’s Stream Run (MR), Hinckley (HI), Chapin (CH), and Swine Creek (SC). Pheretimoid earthworm-invaded sites were: Doan Brook (DB), Bedford (BE), Squire (SQ), and Chagrin River (CR). Two sites: The West Woods (WW) and Holden Arboretum (HA) had both non-invaded and pheretimoid earthworm-invaded areas.
(TIF)

S1 Table Descriptions of field sites in northeastern Ohio. Field sites in northeastern Ohio. Sites were classified as “non-invaded” or “pheretimoid earthworm-invaded” based on presence of large pheretimoid earthworms (or casting material) and the quality of the leaf litter (LL; based on Loss et al. 2013). LL was evaluated at four random locations within the site, each of which was scored from 1–3, with a lower score indicating lower quality. The maximum score a site could receive was a 12 (indicating an intact, healthy forest floor composed of LL from 2+ years), while the minimum score a site could receive was a 4 (indicating a highly disturbed, scant forest floor containing only LL from the previous year).
(PDF)

S1 Video Salamander aggression towards an earthworm during a pilot trial. Pilot feeding trial of the high structure + earthworm treatment. The salamander (*Plethodon cinereus*) exhibits aggressive behavior toward the pheretimoid earthworm, followed by escape behavior. The salamander first displays the “all trunk raised” aggressive posture and then bites the earthworm twice. After the attacks, the salamander quickly moves away from the earthworm and toward the edge of the arena. Lastly, the salamander attempts to find an escape route by poking its snout against the side of the arena. The combination of expensive aggressive behavior toward earthworms and reduced foraging time could result in significant energetic losses for salamanders under natural conditions.
(ZIP)
