# Supplementary figures and images for: Invasive Asian Earthworms Negatively Impact Keystone Terrestrial Salamanders
Source: PLoS One. 2016 May 4;11(5):e0151591. doi: 10.1371/journal.pone.0151591 (PMC4856329; doi:10.1371/journal.pone.0151591)

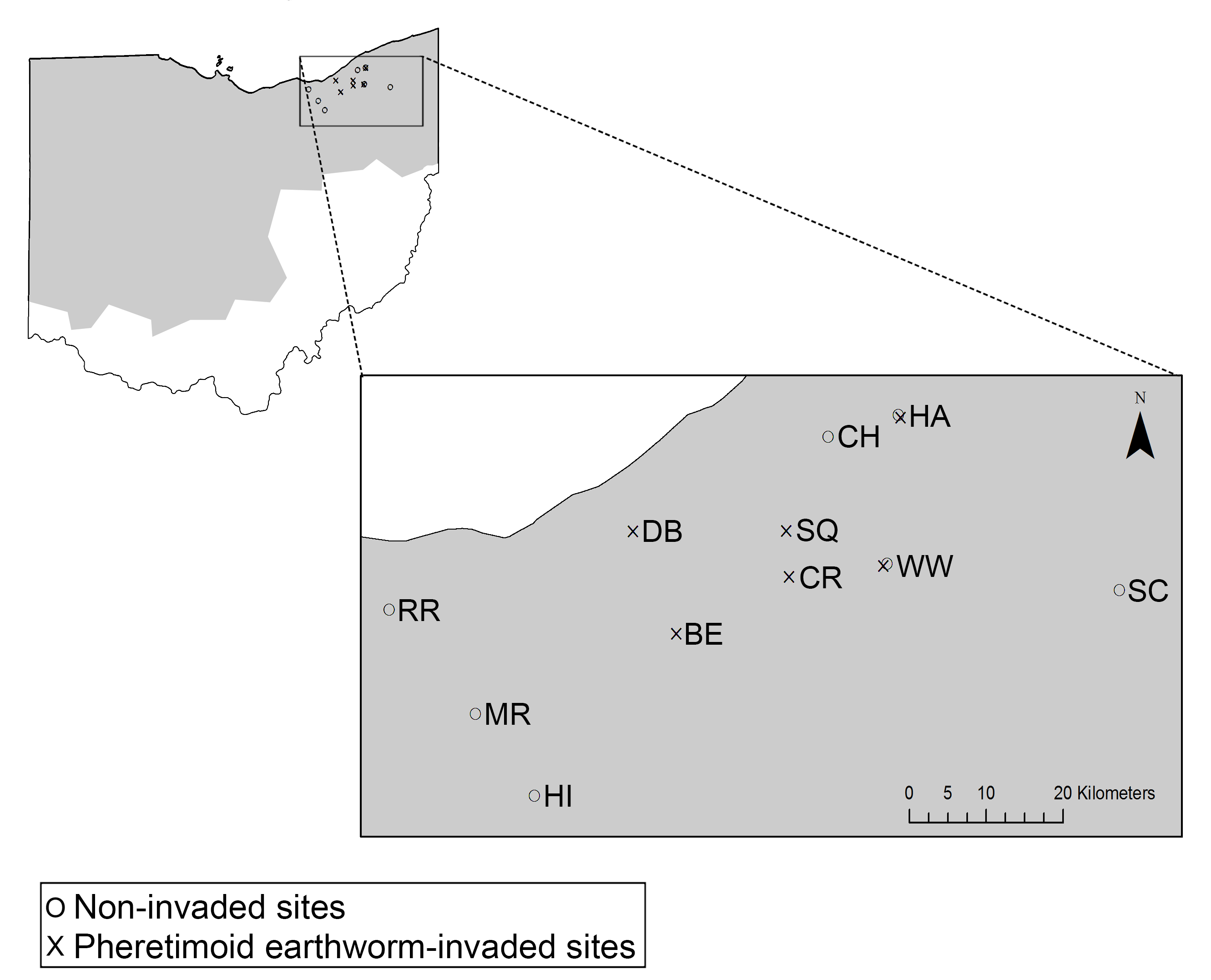

Supplement: S1 Fig — Study site localities in northeastern Ohio. Areas of Ohio denuded by the Laurentide ice sheet during the Wisconsinan glaciation (containing very few North American earthworms) are shaded gray. Non-invaded sites were: Rocky River (RR), Mill’s Stream Run (MR), Hinckley (HI), Chapin (CH), and Swine Creek (SC). Pheretimoid earthworm-invaded sites were: Doan Brook (DB), Bedford (BE), Squire (SQ), and Chagrin River (CR). Two sites: The West Woods (WW) and Holden Arboretum (HA) had both non-invaded and pheretimoid earthworm-invaded areas. (TIF) [file pone.0151591.s001.tif]
