# Supplementary material for: Invasive Asian Earthworms Negatively Impact Keystone Terrestrial Salamanders
Source: PLoS One. 2016 May 4;11(5):e0151591. doi: 10.1371/journal.pone.0151591 (PMC4856329; doi:10.1371/journal.pone.0151591)
Supplement: S1 Table — Field sites in northeastern Ohio. Sites were classified as “non-invaded” or “pheretimoid earthworm-invaded” based on presence of large pheretimoid earthworms (or casting material) and the quality of the leaf litter (LL; based on Loss et al. 2013). LL was evaluated at four random locations within the site, each of which was scored from 1–3, with a lower score indicating lower quality. The maximum score a site could receive was a 12 (indicating an intact, healthy forest floor composed of LL from 2+ years), while the minimum score a site could receive was a 4 (indicating a highly disturbed, scant forest floor containing only LL from the previous year). (PDF) [file pone.0151591.s002.pdf]

**Table S1.**

| | Coordinates | Collection date | # Pheretimoid earthworms | # European earthworms | LL score |
|---|---|---|---|---|---|
| **Non-invaded sites** | | | | | |
| Chapin | 41°35'29.3"N 81°21'55.7"W | 9/18 & 9/25/14 | 0 | 2 | 11 |
| The West Woods | 41°27'36.4"N 81°17'46.0"W | 9/26/14 | 0 | 5 | 11 |
| Hinckley | 41°13'13.9"N 81°42'34.4"W | 10/3/14 | 0 | 16 | 11 |
| Holden Arboretum | 41°36'49.6"N 81°16'59.5"W | 10/4/14 | 0 | 0 | 12 |
| Swine Creek | 41°25'58.4"N 81°01'26.2"W | 10/10/14 | 0 | 0 | 12 |
| Rocky River | 41°24'45.3"N 81°52'46.8"W | 10/17/14 | 0 | 13 | 10 |
| Mill's Run | 41°18'18.3"N 81°46'42.0"W | 10/22/14 | 0 | 0 | 12 |
| ***Pheretimoid earthworm-* invaded sites** | | | | | |
| Doan Brook | 41°29'37.0"N 81°35'37.9"W | 9/25 & 9/30/14 | 50 | 0 | 4 |
| The West Woods | 41°27'27.9"N 81°18'01.8"W | 9/27/14 | 21 | 0 | 6 |
| Holden Arboretum | 41°36'39.6"N 81°16'48.4"W | 10/5/14 | 36 | 1 | 4 |
| Chagrin River | 41°26'47.9"N 81°24'38.9"W | 10/9/14 | 14 | 0 | 4 |
| Bedford | 41°23'15.8"N 81°32'33.9"W | 10/24/14 | 3 | 0 | 9 |
| Squire | 41°29'38.6"N 81°24'50.5"W | 10/25/14 | 5 | 4 | 7 |
